# Gq Signaling in Microglia Triggers Interferon Responses and Improves Outcome After Ischemic Stroke

**DOI:** 10.1002/glia.70209

**Published:** 2026-08-07

**Authors:** Lynn Bitar, Marie‐Luise Brehme, Charlotte Oldenburg, Sara Isla Cainzos, Michael G. Kaul, Tobias Mummert, Malte Borggrewe, Thomas G. Oertner, Tim Magnus

**Affiliations:** ^1^ Department of Neurology University Medical Center Hamburg‐Eppendorf Hamburg Germany; ^2^ Institute for Synaptic Neuroscience, Center for Molecular Neurobiology (ZMNH) University Medical Center Hamburg‐Eppendorf Hamburg Germany; ^3^ Department of Diagnostic and Interventional Radiology and Nuclear Medicine University Medical Center Hamburg‐Eppendorf Hamburg Germany; ^4^ Independent Bioinformatics Consultant Hamburg Germany

**Keywords:** DREADD, interferon‐response, microglia, recovery phase, stroke

## Abstract

Post‐stroke recovery remains limited despite advances in acute reperfusion therapies, underscoring the need to better understand underlying mechanisms that shape repair. Microglia, the resident immune cells of the central nervous system, orchestrate responses to ischemic injury and critically influence neurovascular remodeling, axonal reorganization, and functional recovery. Emerging evidence indicates that inflammatory preconditioning can reprogram microglial responses to subsequent insults, yet the exact intracellular signaling pathways mediating this adaptive state remain incompletely defined. Here, we used a chemogenetic approach to selectively activate Gq signaling in microglia employing a microglia‐specific DREADD mouse model. This strategy mimics Gq‐coupled receptor activation in microglia in the absence of peripheral immune engagement. Pre‐ischemic Gq activation significantly reduced infarct sizes at 24 h after experimental stroke in female mice and at 7 days in both sexes. Morphological analyses revealed that Gq‐conditioned microglia exhibited increased structural complexity, adopting a highly ramified, spatially compact phenotype and higher CD68 expression, indicating increased phagocytic activity. Transcriptional profiling demonstrated that Gq activation primes autophagy‐related defense pathways in microglia, resulting in upregulation of interferon‐stimulated genes 7 days after stroke in both sexes. Together, our findings identify Gq signaling as a key modulatory pathway capable of reprogramming microglial phenotype and enhancing stroke recovery. These results highlight the plasticity of microglial signaling networks and support targeted modulation of microglial Gq pathways.

## Graphical abstract

- Chemogenetic activation of Gq signaling in microglia ahead of ischemic stroke reduces infarct size in mice.
- Seven days after stroke, Gq‐primed microglia in the penumbra show enhanced interferon responses, altered branching patterns, and reduced phagocytosis.
- Female mice subjected to ischemic stroke, showenhanced interferon response after Gq‐priming mediated primarily by invading monocytes.

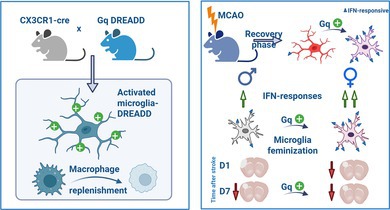

## 1 Introduction

Ischemic stroke is a leading cause of death and disability across the globe, representing more than 70% of stroke incidents (Feigin et al. 2021). This underscores the urgent need for a deeper understanding of the underlying pathophysiological mechanisms to achieve neuronal recovery. It is caused by a cerebral artery occlusion that results in hypoperfusion and subsequent ischemia of the parenchyma supplied by the affected artery, ultimately affecting the function of this part of the brain (Dirnagl et al. 1999; Lipton 1999). Recovery from stroke poses significant challenges in both clinical and experimental settings, requiring a deeper understanding of the interactions between key cellular players (Moskowitz et al. 2010). Microglia are the first responders to ischemic injury, and their activation can influence recovery through phagocytosis and secretion of trophic factors (Jiang et al. 2020; Schafer et al. 2012). In fact, their activation coincides with neuroplasticity after stroke both temporally and spatially (Price et al. 2006; Yu et al. 2021). Microglia establish direct, prolonged contacts with synapses following an ischemic insult (Wake et al. 2009). This interaction and active surveillance has been linked to detrimental or beneficial neuronal activity changes (Umpierre and Wu 2021; Whitelaw et al. 2023). It is to a large extent mediated by microglial G protein coupled receptors (GPCRs) which control microglial activation and polarization phenotype (Zhao et al. 2025). GPCRs are among the largest families of protein receptors in mammals that are essential regulators of numerous physiological and pathological processes (Leysen et al. 2021; Zhang, Chen, et al. 2024). Notably, 20%–50% of currently available drugs target GPCRs, underscoring their potential in drug discovery and development (Rask‐Andersen et al. 2014; Sriram and Insel 2018). To target these GPCRs, we rely on a non‐invasive, chemogenetic approach using Designer Receptors Exclusively Activated by Designer Drugs (DREADD) (Alexander et al. 2009). The most commonly used ligand for these receptors is Clozapine‐N‐Oxide (CNO), a pharmacologically inert metabolite of Clozapine (Armbruster et al. 2007; Urban and Roth 2015).

Activation of microglia is mainly mediated by Gq‐coupled receptors, such as P2Y6 receptors activated by UDP released from stressed or damaged cells, P2Y1 receptors activated by extracellular ATP/ADP, and lipid‐sensing receptors (e.g., LPA3) that detect nerve damage. To selectively activate Gq in microglia, we generated a microglia DREADD mouse line to investigate how microglial priming prior to an ischemic insult affects stroke outcome. Preconditioning can limit tissue damage of subsequent transient ischemia/reperfusion, and various preconditioning strategies, such as hypoxia or inflammatory stimuli, have previously been explored (Helbing et al. 2024). As these approaches induce general inflammatory responses, it is not completely understood how they enhance the tolerance to subsequent acute insults (Zhu et al. 2025). Limiting the manipulation to a single G protein pathway in one specific cell type, we move beyond symptoms to identify the root determinant of neuroprotection following stroke.

## 2 Materials and Methods

### 2.1 Transgenic Mice

Gq‐DREADD mice were generated by crossing B6.129P2(C) – Cx3cr1^(tm2.1/cre/ERT2)Jung/J^ Gt(ROSA)26‐Sor^tm9(CAG‐tdTomato)Hze^ (JAX: 020940; JAX: 007909), which carry a tamoxifen‐inducible Cre‐recombinase in microglia and a floxed fluorescent marker tdTomato, with mice heterozygously carrying the floxed Tg^(CAG‐CHRM3,‐mCitrine)1Ute/J^ (JAX: 026220) allele. This cross generated offspring expressing Gq‐DREADD (CHRM3) and tdTomato in microglia along with littermate controls without Gq‐DREADD. All mice were housed and bred at the University Medical Center Hamburg‐Eppendorf with a 12 h light/dark cycle and had water and food *ad libitum*. All procedures were performed in compliance with German law and the guidelines of Directive 2010/63/EU. The study was approved by the local authorities (Amt für Verbraucherschutz, Lebensmittelsicherheit und Veterinärwesen, Hamburg; permission N075/2023, #103/22, #ORG1106).

### 2.2 Mouse Genotyping

Tail biopsies were taken from mice at postnatal days 3–4 and were lysed in 75 μL lysis buffer containing: 25 mM NaOH, 0.2 mM EDTA‐H2O, H2O for 60 min at 95°C. Subsequently, 75 μL neutralization buffer (40 mM Tris‐HCL, pH 5.5) was added. The lysed samples were then used for a PCR based genotyping using AmpliTaq GoldTm 360 Master Mix (Applied Biosystems) and different primer combinations (Table S1).

### 2.3 Permanent Distal Cerebral Artery Occlusion Model (pMCAO)

Permanent occlusion of the distal MCA was performed as previously described (Llovera et al. 2014). Briefly, mice were anesthetized with isoflurane delivered in 100% O_2_ and placed on their side. After a skin incision between the eye and ear, the temporal muscle was removed and the MCA identified. Next, a burr hole was drilled over the MCA and all three branches of the MCA bifurcation were coagulated through an electro‐coagulator. Body temperature was maintained at 37°C throughout surgery using a feedback‐controlled heating pad. The mice were then kept in their home cage for recovery with facilitated access to water and food. Animals were included if they successfully underwent focal cerebral ischemia induction with complete vessel occlusion. Exclusion criteria were defined a priori and included death during surgery, death within the first 24 h after stroke induction, failure to achieve complete vessel occlusion, and loss of > 20% of body weight. Animals that died during surgery or within the first 24 h after stroke induction were excluded from all functional and histological outcome analyses. One mouse per gender died during surgery and two other mice per gender died within 24 h after surgery. This makes the mortality rate less than 15%. None of the mice lost > 20% of their body weight. Inclusion and exclusion criteria were applied identically to both sexes.

### 2.4 Tamoxifen and CNO Administration

(Z)‐4‐Hydroxytamoxifen (Z‐4‐OHT) (HelloBio, Cat. #: HB2508) was dissolved in corn oil (injection volume: 100 μL per 20 g body weight) and administered in a single intraperitoneal injection at a dose of 1 mg per 20 g body weight to induce Cre‐mediated recombination. The animals were checked daily during the week of injection and weighed additionally during the first 5 days. Clozapine N‐oxide dihydrochloride (CNO; Tocris Bioscience, Cat. #: 6329) was freshly prepared in sterile saline and injected intraperitoneally at a dose of 1.25 mg/kg in a quantity of 0.1 mL/10 g body weight for three consecutive days with the final injection 1 h prior to stroke accumulating both the priming effect of earlier Gq activation and the acute presence of the ligand at the time of injury. Control animals received equivalent volumes of vehicle solution. All injections were performed at consistent times of day to minimize circadian variability.

### 2.5 Magnetic Resonance Imaging in Mice

Scans were acquired with a 7T small‐animal Bruker MRI system (BioSpec 70/30, Bruker) 24 h after stroke using a four‐channel receive head coil. To reduce motion‐related artifacts, animals were kept under isoflurane anesthesia (1.2%–1.8% in 100% oxygen) during the entire data acquisition period. Respiration rate was continuously monitored to ensure physiological stability. A total of 20 animals were examined using MRI 24 h after stroke. The cohort consisted of 9 females and 11 males, distributed across two groups (Veh vs. CNO). Specifically, the Veh group comprised 4 females and 5 males, and the CNO group comprised 5 females and 6 males.

For infarct detection, an axial planned multislice 2D turbo spin‐echo sequence was acquired (TR 5000 ms, TE 58 ms, turbo factor 7, three averages) with a field of view of 20 × 18 mm^2^ and a matrix of 256 × 232. Twenty‐four slices were obtained with a slice thickness of 400 μm and an interslice gap of 100 μm, resulting in a voxel size of 78 × 78 × 500 μm^3^. Following acquisition, all datasets were subjected to quality control procedures.

### 2.6 Infarct Size and Histology

Mice were perfused intracardially with 0.9% PBS followed by 4% paraformaldehyde (PFA). Brains were isolated, fixed overnight, placed in 30% sucrose and then serially sectioned (30‐μm thickness) using a cryostat and distributed across eight slides in sequence. Infarct size was assessed by delineating infarcted regions. In brief, 15 coronal sections per brain were stained with cresyl violet (Carl Roth #2100512). Infarct area was measured on each section using ImageJ and corrected to account for edema. Areas were then integrated to obtain total infarct volume. At this time point after stroke, tissue necrosis and structural fragility are expected to lead to partial tissue loss during sectioning or immunohistochemical processing. DAB staining was done using anti‐iba1 (Wako, Cat.#: 019‐19741) to count microglial cells imaged using Sysmex Pannoramic MIDI II slide scanner. Immunofluorescence was performed using antibodies against Iba1 (Wako, Cat.#: 019‐19741), NeuN (Novus Biologicals, Cat.#: NBP1‐77686) imaged using Zeiss MP platform and we used the Click‐iT Plus TUNEL‐Assay‐Kits for in situ apoptosis detection (ThermoFisher, Cat. #: C10617) to assess dead cells using QuPath software. To minimize sampling bias, we used a systematic random sampling strategy covering the full rostro‐caudal extent of the infarct within the MCA territory. Specifically, every 8th section (30 μm thickness) was collected, and five sections per animal were analyzed, spanning anterior to posterior infarct regions (including striatum, cortical areas, and hippocampal‐adjacent regions). Within each section, six predefined regions of interest (ROIs) were analyzed, resulting in 30 sampling sites per animal. This approach ensured uniform coverage of infarct heterogeneity while avoiding operator‐dependent selection bias. Microglia morphology was analyzed based on the tool and R‐script published by Kim et al. (Kim et al. 2024). All analyses were performed 1 week after the ischemic insult.

### 2.7 Preparation of Hippocampal Slice Cultures

Organotypic slice cultures were prepared from mice at P4‐7 as described (Gee et al. 2017). In brief, newborn mice were anesthetized with 80% CO_2_ 20% O_2_ and subsequently decapitated. Brains were quickly extracted and kept in an ice‐cold dissection medium comprising: 1 mM CaCl_2_, 5 mM MgCl_2_, 10 mM D‐glucose, 4 mM KCl, 26 mM NaHCO_3_, 0.001% phenol red, and 2 mM kynurenic acid. The hippocampi were dissected and sliced into 410 μm sections using a tissue chopper. These slices were then placed on porous membranes (Millicell CM, Millipore). Cultures were maintained at 37°C in a 95% O_2_ 5% CO_2_ atmosphere in a slice culture medium containing 80% Minimum Essential Medium (MEM, Sigma M7278) and 20% heat‐inactivated horse serum (Sigma H1138), supplemented with 1 mM L‐glutamine, 0.00125% ascorbic acid, 0.01 mg/mL insulin, 1.44 mM CaCl_2_, 2 mM MgSO₄, and 13 mM D‐glucose. After the first 24 h, cultures were incubated in 1 μM (Z)‐4‐hydroxy‐tamoxifen (Sigma H7904) added to the slice medium for 24 h to induce Cre‐activation. Slice cultures were used for experiments between 21 and 24 days in vitro (DIV).

### 2.8 Immunohistochemistry

Fixed cultures were blocked for 2 h at room temperature in freshly prepared blocking solution containing 6% normal goat serum and 2% Triton X‐100 in 1× PBS. Subsequently, slices were incubated with primary antibodies diluted in blocking solution at 4°C for 7 days. To assess microglial phagocytic activity, cultures were immunostained against CD68 (Bio‐Rad, #MCA1957, 1:200) and Iba1 (Synaptic Systems, HS‐234013, 1:500). After primary antibody incubation, slices were washed three times in 1 × PBS and incubated with secondary antibodies diluted in blocking solution at 4°C for 3 days (goat anti‐rat IgM Alexa Fluor 647, Invitrogen #A21248, 1:1000; goat anti–guinea pig IgG [H + L] Alexa Fluor 568, Invitrogen #A11075, 1:1000). Prior to mounting, slices were washed three times in 1× PBS and counterstained with DAPI for 10 min. Slices were mounted using Shandon Immuno‐Mount under glass coverslips. Imaging was performed using a confocal laser scanning microscope (Zeiss LSM 900) equipped with a Zeiss C Plan‐Apochromat 63×/1.40 oil objective. For each culture, 2–3 stack were acquired in Airy scan superresolution mode (2048 × 2048 pixels), 0.5 μm z step size, using sequential excitation at 405, 568 and 647 nm. Acquired images were randomly renamed using a custom‐written Fiji script to blind the investigator during analysis in Imaris (10.1). For each microglial cell, a surface was generated based on the Iba1 signal. Within this Iba1‐defined surface, a corresponding CD68 surface was created to quantify the CD68 volume per microglia.

### 2.9 Single Cell Sequencing

Single‐cell transcriptomic profiling was performed using the 10× Genomics Chromium platform (10× Genomics, Pleasanton, CA). Fresh parenchymal tissue isolated 1 week after stroke was rapidly dissected and enzymatically dissociated into a single‐cell suspension using a collagenase/DNase (digestion solution) solution and protease inhibitor mix followed by filtration through a 100 μm strainer to remove debris and cell aggregates. Following a Percoll gradient, cells stained with anti‐CD11b and anti‐CD45 and sorted for microglial cells (CD45 high, CD11b int.). For male mice, a total of two control animals without CNO and three animals with Gq activation were processed. For female mice, a total of two mice per group were used. For sample multiplexing, single‐cell suspensions were incubated with TotalSeq‐B hashtag antibodies (1–4) (BioLegend) according to the manufacturer's protocol, enabling subsequent demultiplexing of pooled samples. Ten thousand cells per sample were loaded onto the Chromium Controller for single‐cell encapsulation and barcoding according to the manufacturer's protocol. cDNA libraries were generated using the Chromium Single Cell 3′ Reagent Kits v3.1 and sequenced on an Illumina NovaSeq 6000 system aiming for 30.000 read pairs per cell. Raw sequencing reads of gene expression and hashtag antibody oligos were processed using the nf core/scRNAseq pipeline (v4.0.0) with Cell Ranger (v8.0.0) for alignment to the 10× Genomics mouse reference genome (GRCm39, release 2024 A). Samples were demultiplexed based on hashtag antibodies using Seurat::HTODemux function (v5.2.1) (Almeida et al. 2025). Downstream analysis was performed using Scanpy (v1.11.1) (Wolf et al. 2018). Low quality cells and putative doublets were removed based on quality control criteria, including a mitochondrial read fraction below 5% and library specific thresholds on the number of detected genes adjusted for sequencing depth. Doublets were further filtered using Scrublet (v0.2.3) (Wolock et al. 2019). Cell numbers before filtering: 3789 for males and 2673 for females; after filtering: 3454 for males, 2285 for females. Counts were normalized using sc.pp.normalize_total followed by log transformation with sc.pp.log1p, and highly variable genes were identified using the Seurat v3 method, selecting the top 2000 genes. Principal component analysis was performed and UMAP embeddings were computed using the first 10 principal components, with male and female samples analyzed separately. Initial Leiden clustering was applied, and low‐quality clusters characterized by low gene counts were removed (fewer than 100 cells in total). Cell numbers after demultiplexing and QC thresholds per sample were as follows: male mice control (no CNO): 179 and 1018 cells, male Gq primed: 588, 726, and 933; female mice control: 597 and 598, female Gq primed: 358 and 662. Leiden clustering was then used to define cell subsets, which were annotated based on known marker genes for microglia subsets and other myeloid cell types. Differential gene expression between CNO and vehicle control conditions was assessed using the MAST framework (v1.32.0) (Finak et al. 2015). Gene set enrichment analysis was performed using GSEApy (v1.0.3) (Fang et al. 2022), with genes ranked by log_2_ fold change from the CNO versus control comparisons. Gene set activity scores for pathway associated gene sets were calculated using AUCell via decoupler (v1.8.0) (Aibar et al. 2017), and differential activity was tested using a Wilcoxon rank sum test. Data was visualized using Seurat. Statistical analysis was performed at the single‐cell level (Tables S2–S12).

### 2.10 Statistical Analysis

Experimental data were analyzed using GraphPad Prism 9 and are presented as means ± SEM. Comparison between groups was performed using a two‐tailed unpaired *t*‐test for normally distributed data or repeated measure for nonparametric data. Asterisks indicate significance with *p < 0.05, **p < 0.01, and ***p < 0.001 for all datasets. Group sizes were determined based on power analysis (G power software) for the primary outcome, infarct volume. Using data from previous similar experimental setups in our lab and published protocols (Szalay et al. 2016), an alpha of 0.05 and Power (1‐β err prob) of 0.8 and an effect size of 1.66 with two groups statistical test: “*t*‐test; Means: difference between two independent means (two groups).” The estimated number of animals required per group was 7. The actual numbers used for infarct volume quantification ranged from 7 to 10 animals per group, exceeding this minimum and ensuring adequate statistical power. For immunofluorescence analyses (TUNEL+/NeuN+ cells, microglial morphology, CD68+ phagosomes), 5–6 animals per group were analyzed. For the cell death assay, 5 systematically distributed sections and 6 ROIs per section were sampled within each animal (30 fields per animal) to capture intra‐animal variability and enhance precision.

## 3 Results

### 3.1 Priming Microglia by Gq Activation Improves Stroke Outcome

**FIGURE 1:**
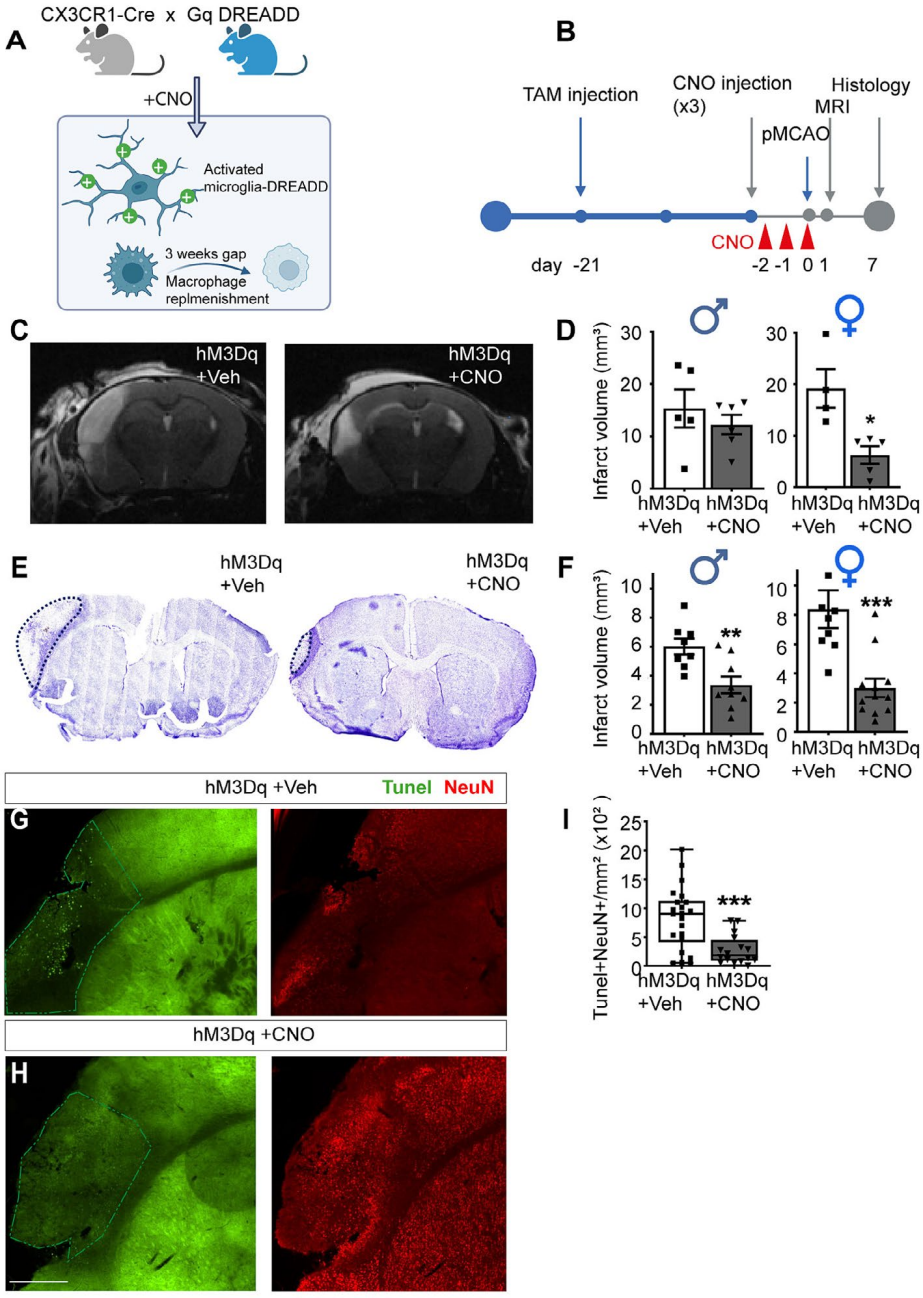
Activation of Gq component after distal medial cerebral artery occlusion improves stroke outcome. (A) Schematic diagram of the generated mouse model by crossing DREADD mice with CX3CR1‐Cre line. Three weeks after tamoxifen injection, circulating macrophages are renewed, preventing their activation following CNO injection. (B) Time line showing tamoxifen injection to activate Cre recombinase and CNO injections on three consecutive days prior to stroke surgery. (C) T2‐weighted MRI measurements 1 day after stroke. (D) Infarct volume in male (Veh, *n* = 5; CNO, *n* = 6) and female (Veh, *n* = 4; CNO, *n* = 5) mice receiving either CNO (gray bars) or a control vehicle (Veh, white bars) (E). Coronal brain sections 7 days after stroke, cresyl violet staining. (F) Infarct volume in male (Veh, *n* = 8; CNO, *n* = 9) and female mice (Veh, *n* = 9; CNO, *n* = 12) 7 days after stroke. (G, H) Representative images of infarct area showing Tunel‐positive cells (green) and NeuN‐positive cells (red) in control (+Veh) and Gq‐activated (+CNO) group. Scale bar: 0.5 mm. (I) Quantification of neuronal cell death (double‐labeled cells). Data are shown as mean ± SEM. Significant differences indicated with * for *p* < 0.05, ** for *p* < 0.005, and *** for *p* < 0.001.

Floxed Gq‐DREADD mice were crossed with the microglia‐specific tamoxifen‐inducible driver line CX3CR1‐Cre^ERT2^. Recombination of the transgene was activated 3 weeks ahead of stroke induction to allow for complete turnover of circulating macrophages (Kellogg et al. 2023). Three days prior to permanent middle cerebral artery occlusion (pMCAO), we injected Clozapine N‐oxide dihydrochloride (CNO) to activate Gq signaling in microglia (Figure 1A,B). Infarct volume was assessed 1 day after pMCAO by MRI (Figure 1C,D) and 7 days later by Nissl staining (Figure 1E). Chemogenetic priming of microglia reduced infarct volume compared to control mice (vehicle group). One day after occlusion, the effect was significant in female mice (Figure 1D). We also determined the apparent diffusion coefficient (ADC) inside the stroke area, which was less depressed in Gq‐primed animals, suggesting less severe tissue damage (Figure S1). During the recovery phase (7 days after stroke), both male and female mice had significantly smaller infarct volumes when microglia were Gq‐primed (Figure 1E,F). This effect was observed consistently across sexes indicating that both female and male microglia exhibit a neuroprotective phenotype when Gq activation is induced 3 days prior to ischemic injury.

Concomitantly, we found strongly reduced cell death in the infarct area in the Gq‐primed group compared to controls as seen by the reduced number of Tunel and NeuN double‐positive cells, confirming the neuroprotective effect of microglia priming (Figure 1G–I). Gq activation did not affect the immune responses and cytokine profile of brain‐infiltrating leukocytes after stroke (Figure S2), suggesting direct protective effects of primed microglia on neurons. The effect of Gq activation on stroke‐induced thigmotaxis did not reach significance (Figure S3). Together, these data demonstrate that Gq pre‐activation in microglia has a protective effect during the recovery phase after stroke with slight gender differences within the acute phase after stroke.

### 3.2 Morphological Analysis of Microglia Shows Increased Microglial Complexity in Gq Activated Stroke Mice

**FIGURE 2:**
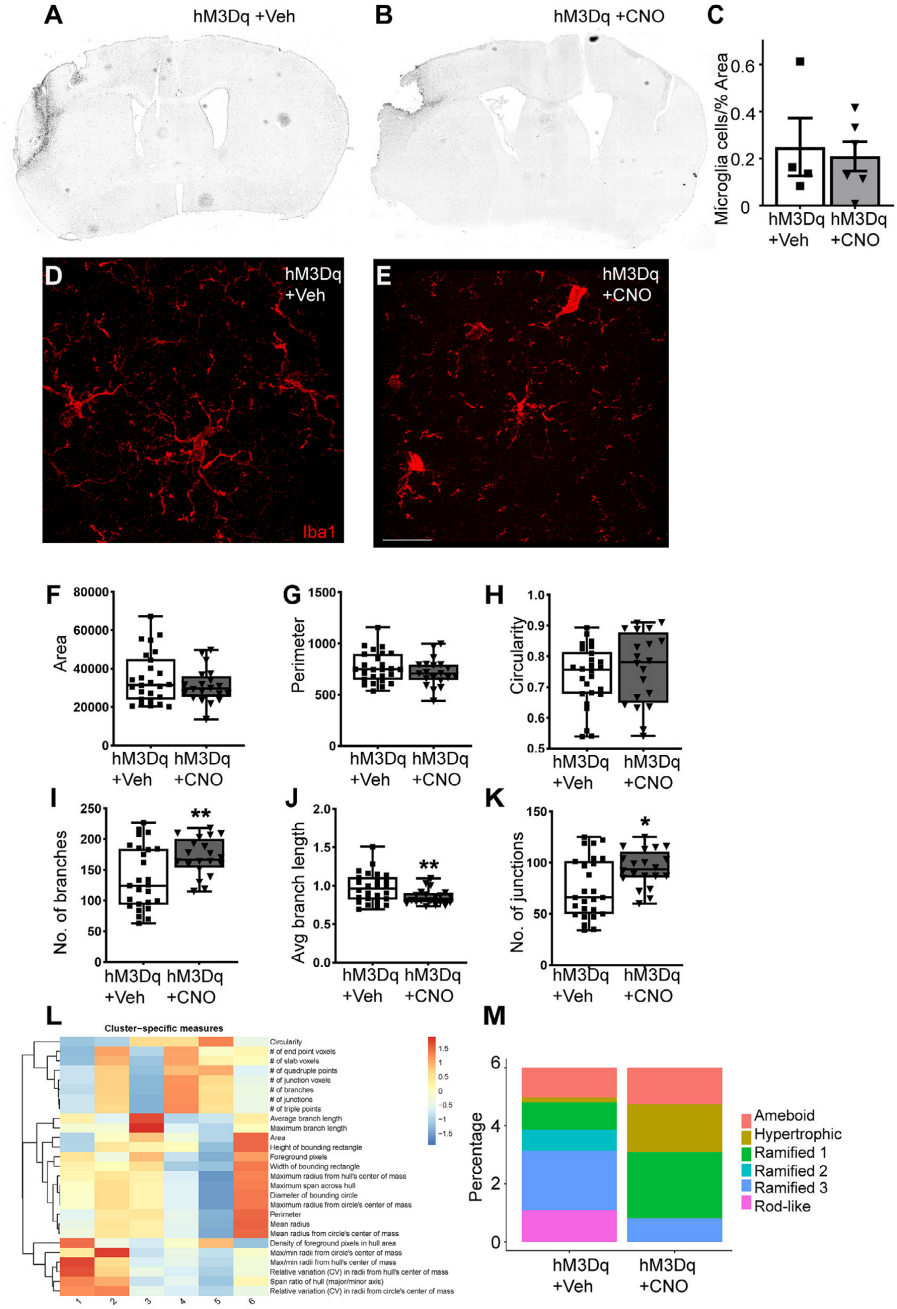
Pre‐activation of Gq affects microglial complexity after stroke. (A–C) Iba1/DAB staining of activated microglia shows no difference in microglial count between control group (+Veh) and Gq‐activated group (+CNO) 7 days after the ischemic insult (Veh, *N* = 4; CNO, *N* = 6 with five slices per mouse and six ROIs in each slice). (D, E) High resolution images of Iba1 immunofluorescence (7 days after stroke) were used for morphometric analysis. (F–H) Parameters related to the size and circularity of microglia cells showed no difference between control (+Veh) and Gq‐activated group (+CNO). (I–K) Microglia branching complexity was higher in the Gq‐activated group (+CNO) along with a reduced average branch length. (L) Heatmap showing cluster specific measures of microglia and (M) the percentage of distribution of different microglial subclusters in control and CNO groups for six mice each. Scale bar: 10 μm. Data are shown as mean ± SEM. Significant differences indicated with * for *p* < 0.05 and ** for *p* < 0.005.

Microglia proliferation following an ischemic insult can be both adaptive and pathogenic and can significantly modify the progression of the disease (Zhang, Li, et al. 2024). Therefore, it is necessary to evaluate if activated microglia replenish the peri‐infarct area (penumbra). Quantification of the density of microglial cells in the penumbra by 3,3′‐Diaminobenzidine (DAB) immunohistochemistry showed no significant effect of Gq priming (Figure 2A–C). This finding prompted us to assess differences in microglia morphology. We analyzed Iba1‐positive microglia (Figure 2D,E) 1 week after stroke in the cortical infarct area in the ipsilateral hemisphere using the open‐source microglia morphology analysis pipeline published by the Ciernia lab (Kim et al. 2024). While the overall soma size (area and perimeter) and gross cell outline were similar between Gq‐activated mice and controls (Figure 2F–H), Gq‐activated microglia had more branches and more branch points (junctions), indicating a more complex arborization (Figure 2I–K). For each identified morphological feature, average values were scaled across clusters (Figure 2L) and were then grouped based on condition into the different microglial morphologies (Figure 2M). Accordingly, we classified cluster 1 as “rod‐like,” Clusters 2, 3, and 6 as “ramified,” cluster 4 as “amoeboid” and Cluster 5 as “hypertrophic.” Remarkably, hypertrophic microglia were at a higher proportion in the Gq‐activated group reflecting enhanced microglial surveillance capacity. This was accompanied by absence of rod‐like microglia in the CNO group pointing to compact, non‐polarized, locally complex and reactive microglial state. Overall, Gq priming does not seem to affect the number, size or diameter of microglia in the penumbra after stroke, but their dense branching pattern hints at an altered functional state. The clustering analysis shows that microglial morphology after activation is consistent with a locally reactive microglial state characterized by enhanced surveillance and adaptive responses.

### 3.3 Gq Activates Defense Responses in Microglia

To assess potential effects of Gq‐activation on the transcriptional response of microglia, we performed single‐cell RNA sequencing. Three days of Gq activation did not shift the relative frequencies of homeostatic versus activated microglia subclusters (Figure S4). At the level of specific genes, we found significant upregulation of Heat Shock Protein Family A Member 8 (Hspa8) and Heat Shock Protein 90 Alpha Family Class B Member 1 (Hsp90ab1) in Gq‐activated microglia, which play key roles in chaperone‐mediated autophagy (Figure S4). Gene set enrichment analysis (GSEA) of microglia (Gq‐activated vs. controls) revealed an enrichment in inflammation/infection related signaling pathways, albeit in the absence of any infectious agent (Figure S4). In summary, Gq activation seems to prepare microglia for enhanced phagocytosis and autophagy.

### 3.4 Elevated Interferon Response in Microglia Contributes to Protection After Stroke

**FIGURE 3:**
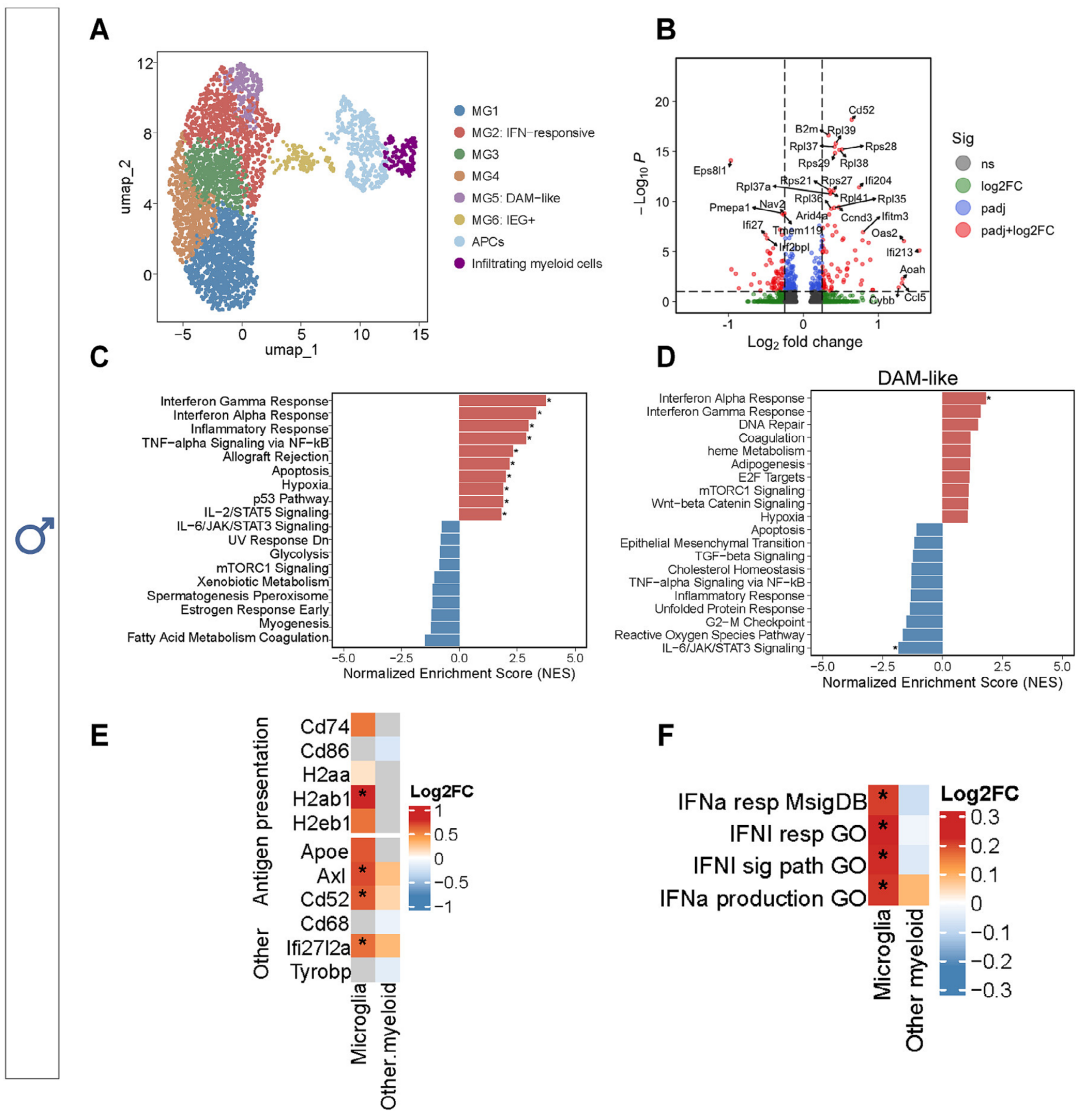
Increased interferon response in both sexes after microglial Gq activation in stroke mice. (A) UMAP plot in males depicting different identified microglial subclusters along with other cells (myeloid and infiltrating monocytes). MG1, MG3, and MG4 were identified as homeostatic markers, MG2: IFN‐responsive, MG5: DAM‐like, MG6: IEG, APC cluster and infiltrating myeloid cells. (B) Differential expression analysis of all microglial clusters shows upregulated interferon related genes in male stroke mice (three biological replicates) in Gq‐activated (+CNO) group which were compared to vehicle‐injected stroked mice (*p*‐adjusted < 0.1; log_2_ fold change > 0.25, two biological replicates). (C) GSEA enrichment analysis indicates increased IFN gamma and alpha responses in the Gq‐activated group versus vehicle (*p‐adjusted < 0.1). (D) GSEA enrichment analysis shows increased IFN‐I response in the DAM‐like cluster. (E) Heatmap depicting log_2_ fold change of selected genes shows elevated antigen expression (*H2‐ab1*) and the interferon‐stimulated gene *Ifi27l2a* in microglia in the Gq‐activated group compared to vehicle control (*p‐adjusted < 0.1; log_2_ fold change > 0.25). Gray tiles indicate low expression of genes in either group, or a small effect size (logFC < 0.1). (F) Heatmap of log_2_ fold changes of gene set activity (AUC) in male mice shows a strongly upregulated IFN response with activated Gq compared to the control group (*p‐adjusted < 0.1; log_2_ fold change > 0.1).

To evaluate modifying effects of Gq‐priming on the transcriptional response of microglia to stroke, we performed single‐cell RNA sequencing 7 days after pMCAO. We identified six different microglial subclusters in males and five in females, along with other myeloid cells. The microglia clusters identified in males included homeostatic clusters (MG1, MG3, MG4), IFN‐responsive cluster (MG2), disease‐associated microglia (DAM‐like) (MG5), and an intermediate early gene (IEG) cluster (MG6) (Figure 3A). Differential expression analysis in male mice revealed 80 differentially expressed genes (DEGs) that were enriched in microglia from CNO‐treated mice (vs vehicle group), while 62 DEGs were depleted (adjusted *p*‐value < 0.1 and log_2_(fold change) > 0.25, Figure 3B). We performed gene set enrichment analysis (GSEA) to find pathways or phenotypes associated with CNO treatment. The molecular signatures database (MSigDB) showed increased interferon responses in male microglia from CNO‐treated mice (Figure 3C). A major contributor to the increased IFN‐I response seems to be the DAM‐like cluster showing increased IFN‐I response in the Gq activated group (Figure 3D). This was further confirmed by analyzing selected genes, showing upregulation of the interferon alpha‐inducible protein 27 like 2 A gene (*Ifi27l2a*) and the beta chain of the MHC Class II (*H2‐Ab1*) in the CNO‐treated group (Figure 3E). Gene set activity analysis confirmed that the enhanced activity of IFN‐related genes was indeed found in resident microglia and not due to invading myeloid cells (Figure 3F).

**FIGURE 4:**
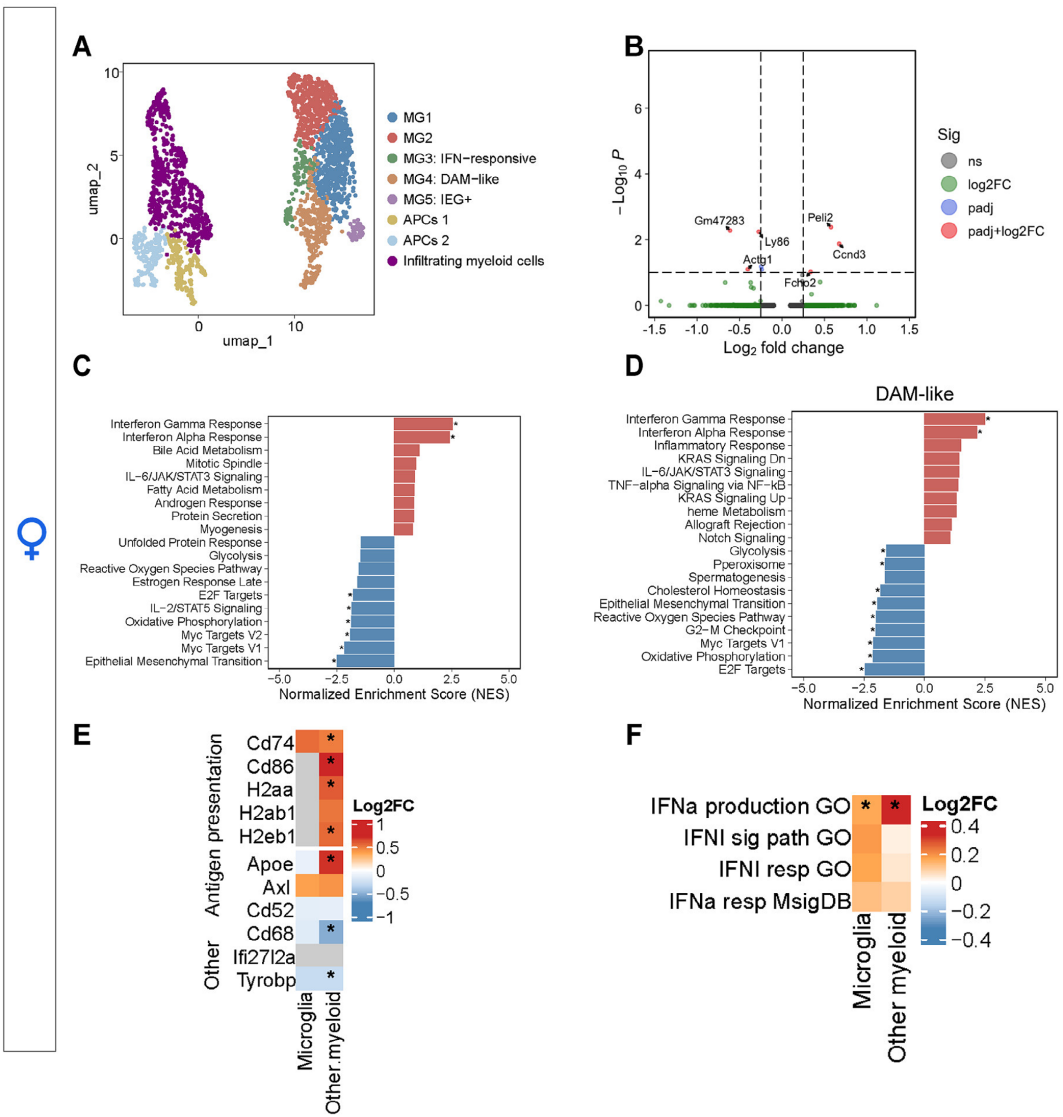
(A) UMAP plot in females showing a similar cluster distribution as males. (B) Female stroke mice (two biological replicates) show a different pattern of differentially expressed genes in the Gq‐activated group compared to controls (two biological replicates) while interferon responses are similarly upregulated. (C) GSEA enrichment analysis indicates increased interferon gamma and alpha responses in female Gq‐activated group vs. vehicle (*p‐adjusted < 0.1). (D) GSEA enrichment analysis shows increased IFN‐I and II response in the DAM‐like cluster. (E) Heatmap depicting log_2_ fold change of selected genes shows mostly changes in myeloid subpopulation in the Gq‐activated group compared to vehicle control (*p‐adjusted < 0.1; log_2_ fold change > 0.25). (F) Heatmap of log_2_ fold changes of gene set activity (AUC) in female mice shows an upregulated IFN response in the activated Gq group compared to the control (*p‐adjusted < 0.1; log_2_ fold change > 0.1). All readouts were done 7 days after stroke. AUC, area under the curve; NES, normalized enrichment score.

Female microglia had a similar cluster distribution (Figure 4A). The effect of Gq priming on the transcriptional profile of microglia from female mice was less pronounced (Figure 4B), but pathways related to IFN responses were similarly upregulated in the CNO‐treated group (Figure 4C). DAM‐like population was also a contributor to the increased IFN‐I and II responses seen in females (Figure 4D). However, in female mice, a considerable part of this IFN response was mediated by infiltrating myeloid cells (Figure 4E). Stroke‐triggered infiltration was strongly boosted by Gq activation in female but not in male mice (Figure S5). Importantly, peripheral immune cells do not express Gq DREADD in our mouse model, suggesting that microglia‐secreted cues were able to attract invading immune cells in female mice. Our analysis revealed that the remodeling of microglial phenotype via Gq DREADD activation is more pronounced in male animals, even though reduced stroke size was detected in both sexes. Microglial priming is associated with elevated IFN responses at 1 week following ischemia, with Gq activation enhancing this response in both sexes.

### 3.5 Sex‐Biased Immune Responses in Primed Microglia Are Regulated by Interferon Responses

**FIGURE 5:**
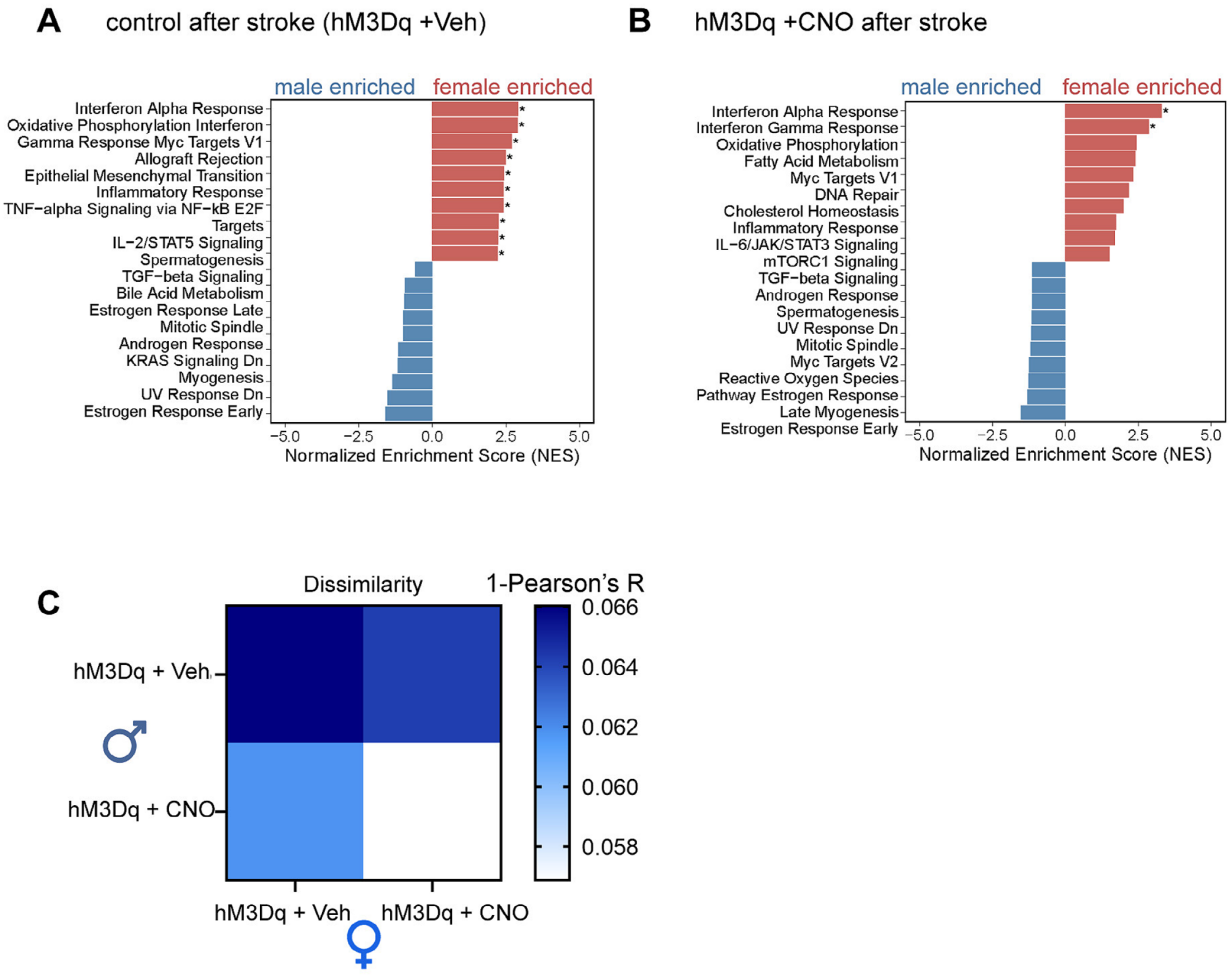
Sex differences in the interferon response of microglia after stroke. (A) Comparing microglia expression profiles in female versus male mice (2 mice per sex) 7 days after stroke shows a markedly stronger response in females. (B) Gq activation before the stroke reduced the sex difference of the microglia response 7 days after stroke (2 female, 3 male mice). (C) Dissimilarity analysis based on gene expression profiles and correlation of these profiles. A higher value corresponds to more dissimilarity. NES, normalized enrichment scores; MSigDB, molecular signatures database (NES > 0 indicates enrichment at the top of the list, NES < 0 indicates enrichment at the bottom of the list). Asterisks next to bars indicate a significant adjusted *p*‐value based on *p*‐Threshold. Threshold for *p*‐Value: 0.1.

Sexual dimorphism in neuroinflammatory responses between males and females after ischemic injury has recently gained more attention (Kerr et al. 2019; Wilson et al. 2013). Preclinical studies have shown that microglia contribute to the sexual dimorphism observed in stroke (Villa et al. 2018). Consequently, we compared transcriptomic profiles of male and female microglia after pMCAO that showed significantly stronger elevated interferon responsiveness in female microglia (Figure 5A). Gq priming reduced the female bias in the stroke response (Figure 5B), leaving IFN responses as the only pathway significantly enriched in female versus male microglia. Dissimilarity analysis based on gene expression profiles and correlation of these profiles shows that primed males show a subtle, but relevant reduction in dissimilarity with females after CNO activation (lower 1‐Pearson's R) (Figure 5C). In conclusion, Gq priming in male microglia alters their genomic profile, promoting responses associated with a protective phenotype.

### 3.6 Gq Priming of Microglia Reduces Phagocytic Capacity After Ischemic Insult

**FIGURE 6:**
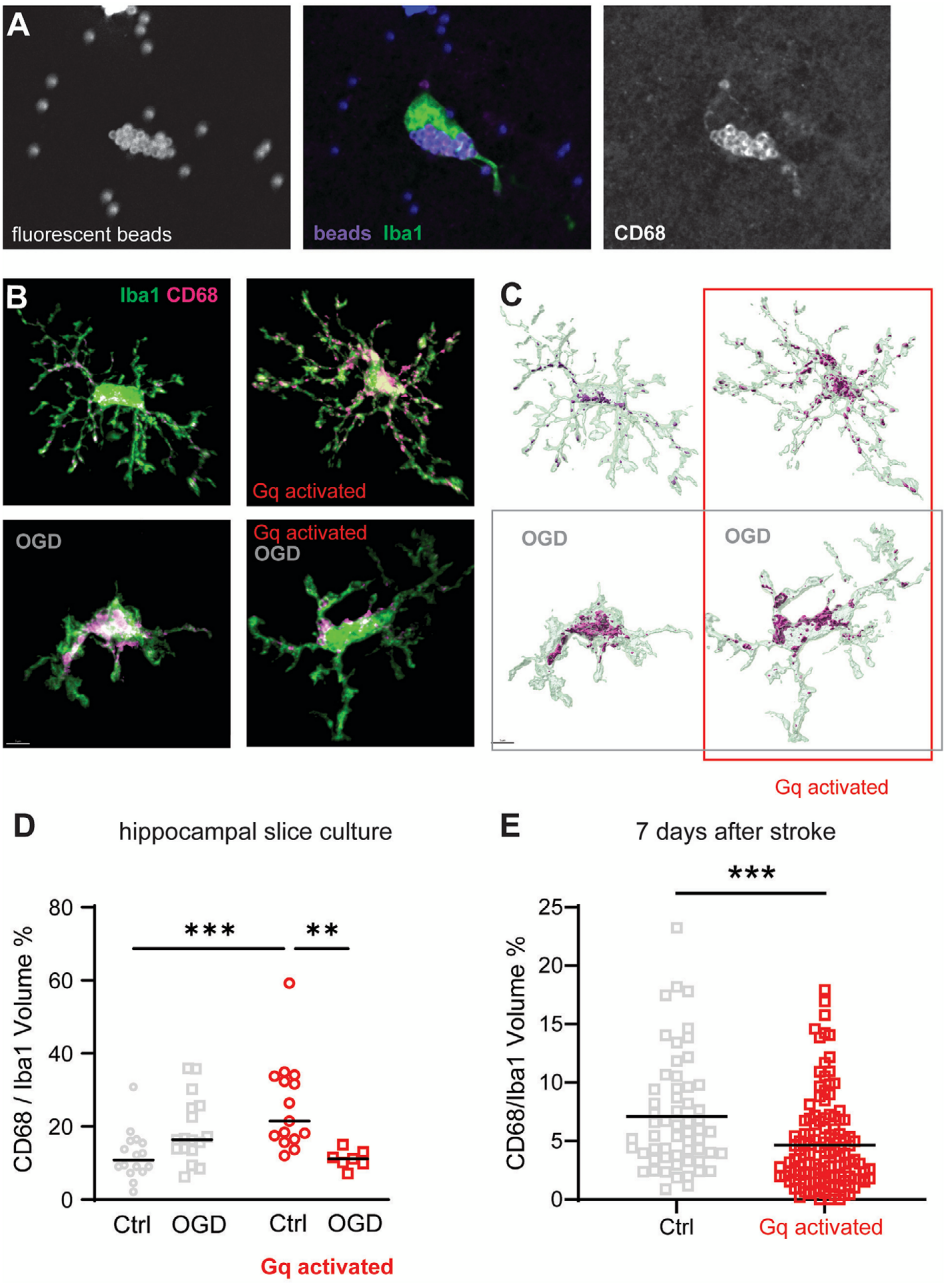
Gq activation in microglia reduces phagocytosis under hypoxic conditions. (A) Phagosomes in microglia (Iba1, green) are selectively labeled by CD68 antibodies in hippocampal slice culture. (B) Examples of CD68 immunofluorescence (magenta/white) in microglia expressing GFP (left) or GqDREADD (right). Lower row: after 20 min OGD. (C) Volumetric analysis (Imaris) of the cells shown in B. CD68 volume is shown in magenta. Scale bars: 5 μm. (D) In naive cultures (Ctrl), Gq activation increased the volume fraction of CD68 (2‐way ANOVA followed by Tukey's multiple comparisons test, **p < 0.005, ***p < 0.001). In Gq‐activated microglia (red), OGD reduced the CD68 volume fraction. *n* = 16, 17, 15, 7 cells. Control group consists of GFP‐expressing microglia and DREADD‐expressing microglia without CNO activation. Bars show mean. (E) Volumetric analysis of microglia in the penumbra 7 days after pMCAO. Gq pre‐activation reduced the CD68 volume fraction (*n* = 58, 121 cells. Two‐tailed *t*‐test with Welch's correction, ***p = 0.001). Bars show mean.

Microglia are the professional phagocytes of the brain, capable of engulfing cell debris and clearing necrotic tissue and apoptotic cells in cerebral diseases (Wang et al. 2021). The phagocytic properties of microglia lie at the intersection of damage, inflammation and repair, and shape recovery. To study the effects of Gq preconditioning on phagocytosis, we stained for CD68, a marker for phagosomes in microglia in organotypic slice cultures (Figure 6A). Oxygen/glucose deprivation (OGD) of hippocampal slice cultures triggered rapid retraction of microglial processes (Figure 6B). To compare the phagosome volume to the total volume of individual microglia cells, we performed 3D volume reconstructions (Figure 6C). As previously established (Churchward et al. 2018), we found that OGD increased phagocytosis compared to control conditions (Figure 6D). Gq‐activation by itself strongly increased phagocytosis, but in combination with OGD, resulted in weak phagocytic activity, comparable to baseline levels (Figure 6D). Next, we performed the same blinded volumetric analysis on microglia in the penumbra, 7 days after pMCAO. Microglia that were pre‐conditioned by Gq activation before the stroke expressed significantly less CD68 (Figure 6E). Thus, it appears that in response to ischemic insult, Gq priming reduces, not enhances, the phagocytic activity of microglia.

## 4 Discussion

Our study demonstrates that targeted manipulation of microglial activation prior to an ischemic injury can safeguard the brain against subsequent damage. A pMCAO induces morphologic and spatial changes in microglia (Del Águila et al. 2024). This complexity reflects the ability of microglia to adopt diverse, dynamic states in response to stress as they develop heterogeneous signatures highly specific to the individual disease environment (Masuda et al. 2020; Wishart et al. 2023). Microglia influence recovery after stroke, and their ability to switch their phenotype can affect the restoration of brain function. Priming the immune system by inflammatory agents can enhance or attenuate the immune response to a secondary insult. Inflammatory preconditioning alters the proteomic response of microglia in cerebral ischemia and correlates with neuroprotective microglial reprogramming (Norden et al. 2015). However, the mechanism of this neuroprotection has been difficult to pin down, as both peripheral and brain‐resident immune cells are activated in these paradigms. Emerging evidence suggests that different types of stimuli that trigger preconditioning confer neuroprotection through a common process which depends on a fundamental genomic reprogramming of the response to injury (Stenzel‐Poore et al. 2007). Among potential targets, GPCRs stand out as accessible receptors for enabling preconditioning responses prior to stroke (Girish et al. 2024). There are reports showing that a subfamily of GPCR is directly involved in preconditioning and microglial polarization (Franco et al. 2021). Preconditioning through GPCR manipulation has also been reported in the heart, supporting a conserved role in regulating protective and adaptive cellular responses beyond the brain (Tong et al. 2004; Xin et al. 2012). Here, we use a chemogenetic approach to selectively activate the Gq pathway only in microglia prior to pMCAO, in the absence of any circulating pro‐inflammatory agents such as LPS. This system enables a controlled and cell‐type–restricted activation of a canonical intracellular pathway that cannot be selectively and temporally matched by currently available physiological stimuli. Our data show that targeted pre‐activation of microglia prior to stroke is a protective strategy, resulting in improved outcome in both male and female mice at a defined pre‐conditioning interval. Gq preconditioning by itself activated autophagy pathways (Figure S4) and endocytic activity (Figure 6), while after ischemia, it reduced endocytosis and strongly enhanced interferon signaling pathways. Furthermore, we show that female microglia, which mount a more robust immune response after stroke, are affected differently by Gq preconditioning than male microglia. It could be argued that Gq priming reduces sex differences in the immune response after stroke by shifting male microglia towards a more protective expression profile. A caveat of the CX3CR1‐CreERT2: R26‐LSL‐Gq‐DREADD model is the haploinsufficiency at the CX3CR1 locus, potentially blunting fractalkine signaling and microglial proliferation as reported in a stroke model (McDonough et al. 2020). While our DREADD activation (CNO dosing) elicited robust Gq effects independent of baseline CX3CR1, future studies using other microglia‐specific Cre lines are needed.

IFN responses are central to the antiviral defense, and some subtypes act as mediators of central nervous system (CNS) inflammation during autoimmunity (Berg et al. 2017; Guo et al. 2008; Khorooshi et al. 2015, 2013; Prinz et al. 2008). They are classified according to their structural homology and receptor type as type I (mainly α and β), II (γ), and III (Kopitar‐Jerala 2017; Wanve et al. 2019) and they play a multi‐faceted role in ischemic injury (Zhang et al. 2017). It has been reported that applying systemic IFN‐β post‐ischemia helps in attenuating brain infarct progression (Veldhuis et al. 2003). Their influence extends to brain function and behavior (Lopez‐Atalaya and Bhojwani‐Cabrera 2025; Roy and Cao 2022, 2020). Females typically mount stronger type I IFN (IFN‐I) responses, that is, more activated innate immune pathways, and are less susceptible to viral infections compared to males, showing that IFN‐I contribute to sexual dimorphism seen in defense responses (Pujantell and Altfeld 2022; Shay 2023). Large‐scale RNA sequencing studies have shown that IFN pathways, in particular IFN‐I responses, are activated after stroke, suggesting that IFN‐I signaling is a hallmark of the differential response in stroke (Androvic et al. 2020). Transient ischemia in male mice induces robust type I IFN‐stimulated genes in microglia 3 days after stroke (McDonough et al. 2017). Our data indicate that 7 days after pMCAO, IFN‐II responses are also involved, which could be secondary to an earlier IFN‐I response. However, the substantial overlap between type I and type II IFN–responsive gene sets complicates the interpretation of pathway enrichment analyses. The critical role of IFN‐I signaling for ischemic preconditioning‐mediated neuroprotection in various mouse models of ischemic injury is well documented (Hamner et al. 2015; Marsh et al. 2009; McDonough and Weinstein 2016; Stevens et al. 2011). LPS preconditioning, for instance, causes genomic changes characterized by a robust cytokine response, involving mainly genes associated with neuroprotective type I interferons (Stenzel‐Poore et al. 2007). Our findings show that in ischemic conditions, the pre‐activation of Gq coupled pathways in microglia leads to an enhanced IFN‐response and neuroprotection. During development, a type I IFN–responsive subset of microglia mediates neuronal engulfment in the mouse cortex and is required for proper cortical development and sensorimotor function (Escoubas et al. 2024). After stroke, preventing the accumulation of damaged neurons in the penumbra is a major task for microglia, which may explain the connection between boosted IFN response and reduced stroke size.

Interferons are typically induced downstream of Toll‐like receptors (TLR) activation by extracellular ligands enhancing antigen presentation (Wanve et al. 2019). TLRs play a central role in virus‐induced immune responses and function as key components of innate immunity by detecting signals of cellular damage as well as invading pathogens (Stevens et al. 2011). TLR ligands have demonstrated remarkable effectiveness in inducing ischemic tolerance (Hua et al. 2008; Stevens et al. 2008, 2014; Tasaki et al. 1997). The TLR9 ligand has demonstrated strong efficacy in a clinically relevant non‐human primate model of experimental stroke (Bahjat et al. 2011). Toll‐like receptor 4 signaling has been implicated in ischemic preconditioning by activating nuclear factor‐kappa B (NF‐κB) and other mediators such as iNOS or COX‐2 inducing an endogenously neuroprotective phenotype (Hamner et al. 2015; Pradillo et al. 2009; Vartanian et al. 2011). TLR7 preconditioning elevates the expression of IFN‐Is (essentially IFNα), providing a path towards neuroprotection in ischemic stroke (Leung et al. 2012). Mice pretreated with a TLR7 agonist prior to MCAO elicit significantly reduced infarct volumes and less pronounced sensorimotor deficits. The reduced damage is associated with upregulation of IFN‐associated genes (*Usp18*, *Oasl2*, *Isg15*, *Ifit1*) that was not evident in the non‐preconditioned group (Leung et al. 2012). TLRs certainly played a role in our Gq priming experiments. Likely, the sex‐dependent different composition of monocyte cell population found in the ischemic brain would have yielded different TLR families and had an impact on the outcome of the different groups. However, rather than acting within the canonical TLR–IFN axis, Gq signaling appears to represent an entry point into a convergent microglial reprogramming state associated with ischemic tolerance.

We acknowledge the limitations of our study, including the evaluation of a highly kinetic microglial phenotype at a single time point in a pathology like stroke, as well as the reliance on one stroke model in young mice, which captures only a subset of stroke patients. We hope that our strategy of chemogenetic intervention will pave the way to investigations of different stroke models, pre‐activation regimes, and aged animals. The sex‐specific differences in stroke outcome we report are supported by evidence from many clinical and experimental studies (Banerjee and McCullough 2022; Ugidos et al. 2022). In a study where microglia from female mice were transplanted into male mice, the progression of post‐stroke injury was minimized independently from hormonal cues, suggesting that microglia maintain their sex‐specific features (Lenz et al. 2015; Roy‐O'Reilly and McCullough 2014; Villa et al. 2018). NF‐κB (nuclear factor kappa B) is transcriptionally more activated in male microglia compared with female microglia, suggesting that male microglia cells were more poised to inflammatory reactions than female microglia (Villa et al. 2018). In fact, we only detect upregulated TNF‐α signaling via NF‐κB in male sorted microglia. An understanding of sex‐specific differences in microglial modulation of the peri‐infarct cortex during post‐stroke repair may reveal novel therapeutic targets to promote long‐term functional brain recovery (Ugidos et al. 2022). Sex‐specific differences in stroke may influence treatment strategies for both males and females. To identify the endogenous molecular mechanisms that govern neuroprotection, cell‐specific priming methods are a valuable approach.

## Author Contributions

**Lynn Bitar:** methodology, formal analysis, investigation, visualization, writing – original draft, writing – review and editing. **Marie‐Luise Brehme:** formal analysis, investigation, visualization, writing – review and editing. **Charlotte Oldenburg:** investigation, visualization, writing – review and editing. **Sara Isla Cainzos:** formal analysis. **Michael G. Kaul:** formal analysis. **Tobias Mummert:** investigation, visualization. **Malte Borggrewe:** formal analysis, visualization. **Thomas G. Oertner:** conceptualization, supervision, funding acquisition, writing – review and editing. **Tim Magnus:** conceptualization, supervision, funding acquisition, writing – review and editing.

## Funding

This work was supported by the German Research Foundation DFG through SPP2395 “Local and peripheral drivers of microglia diversity and function” (T.M. & T.G.O.) and ERC Synergy grant #951515 “Microglia control of physiological brain states” (MICRO‐COPS) (T.G.O.).

## Ethics Statement

All experimental procedures involving animals were approved by the local authorities (Behörde für Justiz und Verbraucherschutz der Freien und Hansestadt Hamburg, approval numbers N075/2023, #103/22, #ORG1106) and were conducted in accordance with national and international guidelines for animal research.

## Conflicts of Interest

The authors declare no conflicts of interest.

## Supporting information


**Data S1:** glia70209‐sup‐0001‐Supinfo1.xlsx.

**Data S2:** glia70209‐sup‐0002‐Supinfo2.pdf.

**Figure S1:** Microglia Gq activation alleviates stroke‐induced restriction of diffusion. (A) Representative maps of the apparent diffusion coefficient (ADC) of both sexes and treatment paradigms, 24 h after pMCAO. Scale bar = 10 mm. (B) 24 h after stroke, mean ADC values inside the stroke area were significantly higher in male (*n* = 11) compared to female mice (*n* = 9), indicating less severe tissue damage. *p* = 0.04 (*). (C) Microglia Gq activation reduced restricted diffusion in comparison to control (Veh, *n* = 9; CNO, *n* = 11), *p* = 1.16 * 10^−6^ (***).

**Figure S2:** Microglial Gq pre‐activation does not affect immune cell infiltration 7 days after stroke. (A, B) Gating strategies of lymphocytes and living cells followed by gating of CD45‐positive lymphocytes and from that the percentage of microglia, B cells and dendritic cells (DC) as well as other microglia‐secreted cytokines in Veh (A) and Gq‐activated (B) groups. (C) Flow cytometric analyses of brain‐infiltrating leukocytes in the ipsilateral hemisphere of male mice show no differences in B cells or dendritic cells (DCs) between control and Gq‐activated groups (Veh, *n* = 4–8; CNO, *n* = 5–9). (D) In male mice, cytokines secreted by microglia were not affected by Gq pre activation (Veh, *n* = 3–6; CNO, *n* = 3–9). (E) No difference in immune infiltration of B cells and DCs were detected in female mice (Veh, *n* = 3–7; CNO, *n* = 6–11) 7 days after stroke. (F) In female mice, cytokines secreted by microglia were not affected by Gq pre‐activation (Veh, *n* = 3–7; CNO, *n* = 3–8).

**Figure S3:** Effects of microglia Gq activation on thigmotaxis after pMCAO. (A) Track plots showing the movement of two mice in the open field arena during 10 min of exploration. (B) Time spent in the center versus the periphery of the arena as a measure of anxiety, evaluated at baseline (BL) and at days 1, 3 and 7 after stroke (male mice). (C) Same analysis for female mice. Microglia Gq activation (light gray) had no effect by itself (BL) and did not significantly alter stroke‐induced thigmotaxis in either sex.

**Figure S4:** Gq activates defense responses in microglia. (A) UMAP‐based clustering of microglia from Gq mice and controls (*N* = 2, 2) after 3 daily CNO injections (no stroke). (B) Microglia clusters (MG1‐5) express the marker genes P2ry12, Tmem119, and Cx3cr1. Clusters MG4 and MG5 express the activation markers interleukin 1 (Il1) and tumor necrosis factor (Tnf). (C) Microglia Gq activation in vivo did not induce state switching. (D) Gq activation lead to significant upregulation of Heat Shock Protein Family A Member 8 (Hspa8) and Heat Shock Protein 90 Alpha Family Class B Member 1 (Hsp90ab1) which play key roles in chaperone‐mediated autophagy. (E) Gene set enrichment analysis (GSEA) of microglia (Gq‐activated vs. controls). Asterisks next to bars indicate a significant adjusted *p*‐value. MG, microglia; BAMs, border‐associated macrophages.

**Figure S5:** Increased stroke‐induced myeloid infiltration in female mice after Gq activation. (A, B) Gating strategy for CD45highCD11bhigh myeloid cells (Veh, *n* = 5 female mice; CNO, *n* = 7 female mice). (C) In female mice, microglia Gq activation leads to increased infiltration of myeloid cells into the ipsilateral hemisphere 7 days after stroke. (D) In male mice, microglia Gq activation has no effect on stroke‐induced myeloid infiltration (Veh, *n* = 7; CNO, *n* = 8). (E) Gene set activity of myeloid subsets show reduced Anxa2 in all female subsets, and (F) increased IFN production in border associated macrophages (BAMs) and IFN‐responsive MHCII macrophages subclasses. Data are shown as mean ± SEM. Significant differences indicated with * for *p* < 0.05, ** for *p* < 0.01.

## Data Availability

The data that support the findings of this study are available from the corresponding author upon reasonable request. The datasets generated during the current study will be deposited to NCBI GEO (https://www.ncbi.nlm.nih.gov/geo/) at the time of publication.
