# Supplementary material for: Gq Signaling in Microglia Triggers Interferon Responses and Improves Outcome After Ischemic Stroke
Source: Glia. 2026 Aug 7;74(10):e70209. doi: 10.1002/glia.70209 (PMC13451549; doi:10.1002/glia.70209)
Supplement: Supplementary file 1 [file GLIA-74-0-s001.pdf]

Supplemental Figures and Table

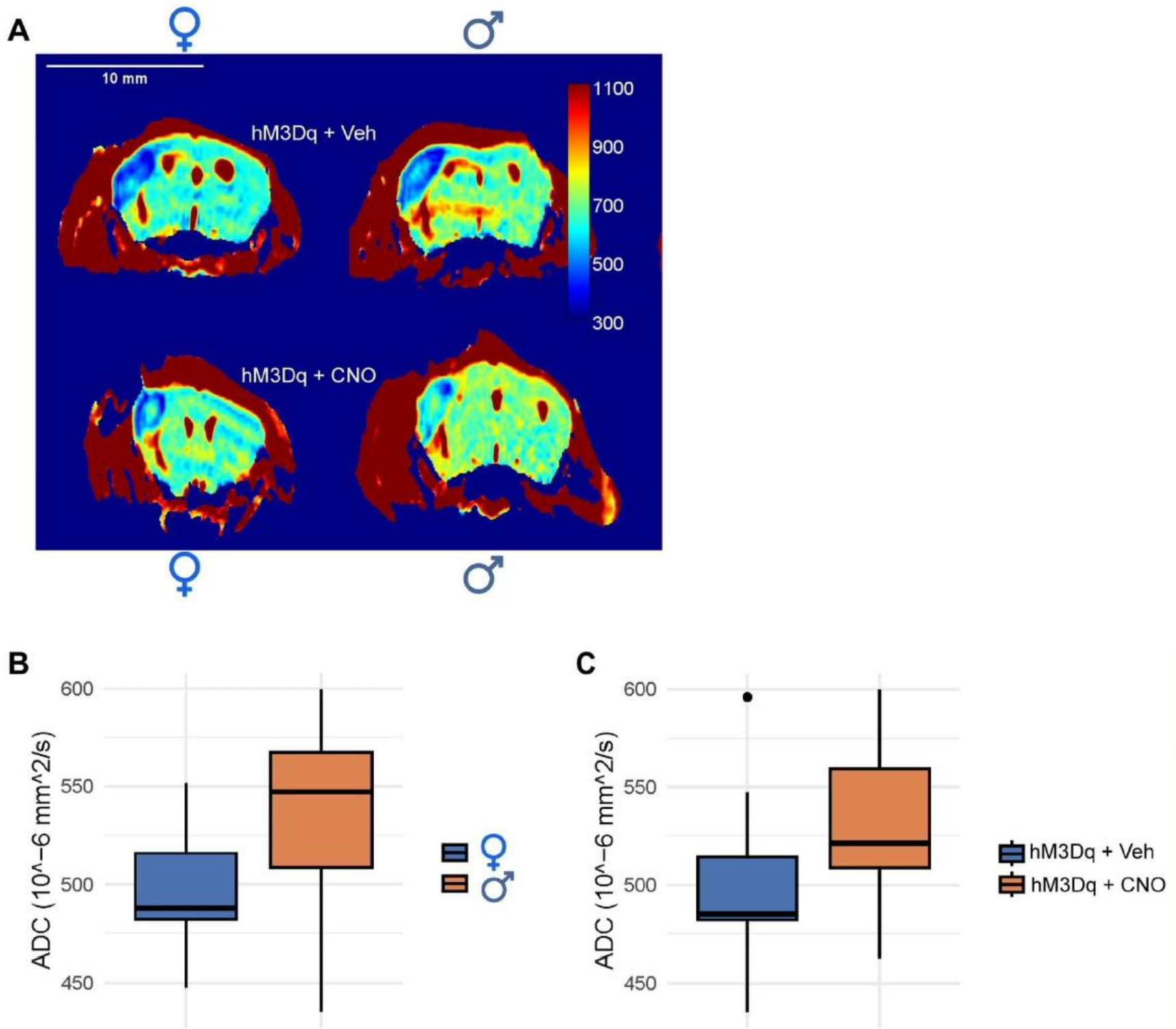

Fig. S1: Microglia Gq activation alleviates stroke-induced restriction of diffusion.

(**A**) Representative maps of the apparent diffusion coefficient (ADC) of both sexes and treatment paradigms, 24 h after pMCAO. Scale bar = 10 mm. (**B**) 24 h after stroke, mean ADC values inside the stroke area were significantly higher in male (n = 11) compared to female mice (n = 9), indicating less severe tissue damage. p = 0.04 (*). (**C**) Microglia Gq activation reduced restricted diffusion in comparison to control (Veh, n = 9; CNO, n = 11), $p = 1.16 * 10^{-6}$ (***).

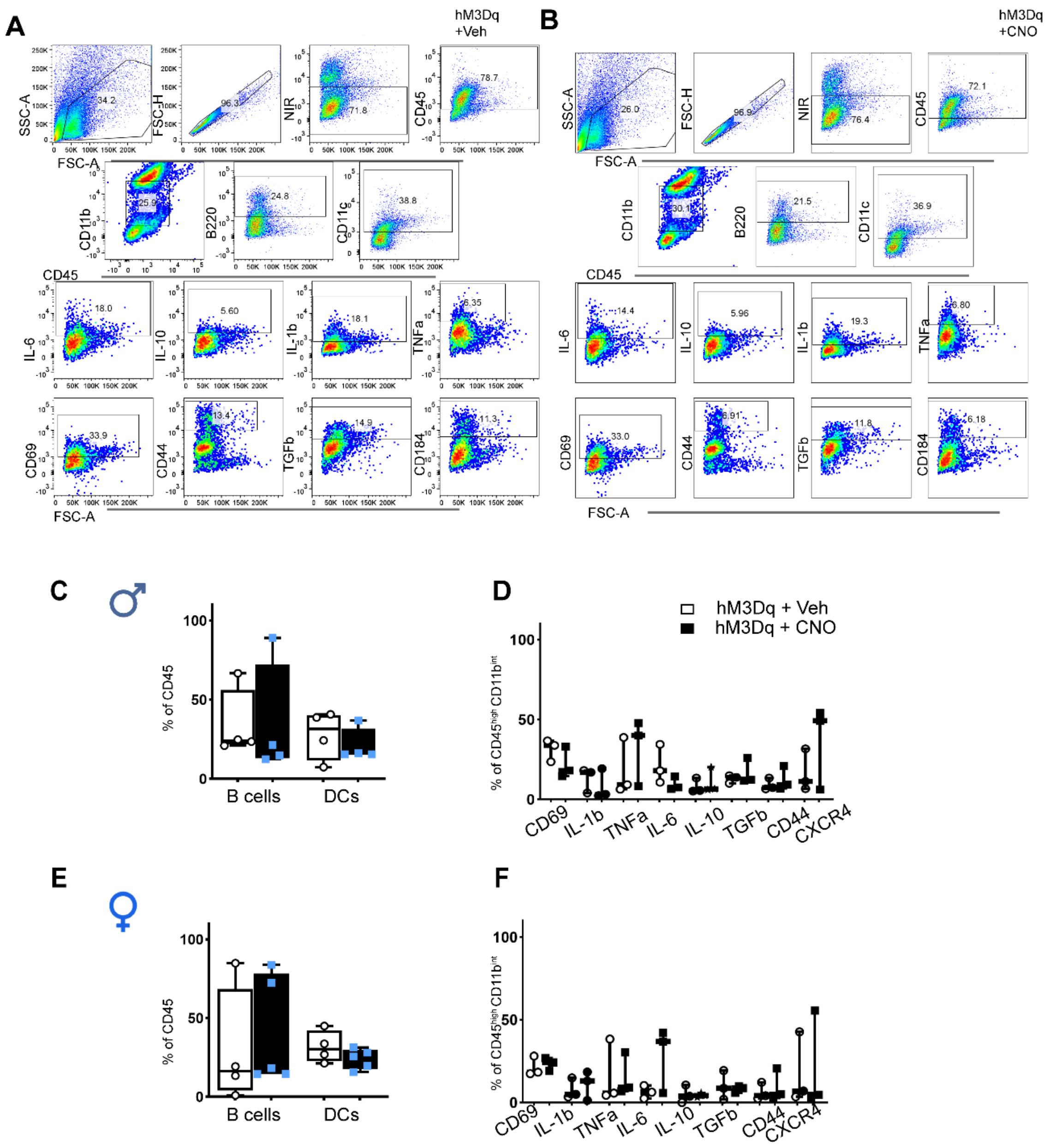

Fig. S2: Microglial Gq pre-activation does not affect immune cell infiltration 7 days after stroke.

(**A-B**) Gating strategies of lymphocytes and living cells followed by gating of CD45-positive lymphocytes and from that the percentage of microglia, B cells and dendritic cells (DC) as well as other microglia-secreted cytokines in Veh (A) and Gq-activated (B) groups. (**C**) Flow cytometric analyses of brain-infiltrating leukocytes in the ipsilateral hemisphere of male mice show no differences in B cells or dendritic cells (DCs) between control and Gq-activated groups (Veh, n = 4-8; CNO, n = 5-9). (**D**) In male mice, cytokines secreted by microglia were not affected by Gq pre-activation (Veh, n = 3-6; CNO, n = 3-9). (**E**) No difference in immune infiltration of B cells and DCs were detected in female mice (Veh, n =3-7; CNO, n = 6-11) 7 days after stroke. (**F**) In female mice, cytokines secreted by microglia were not affected by Gq pre-activation (Veh, n = 3-7; CNO, n = 3-8).

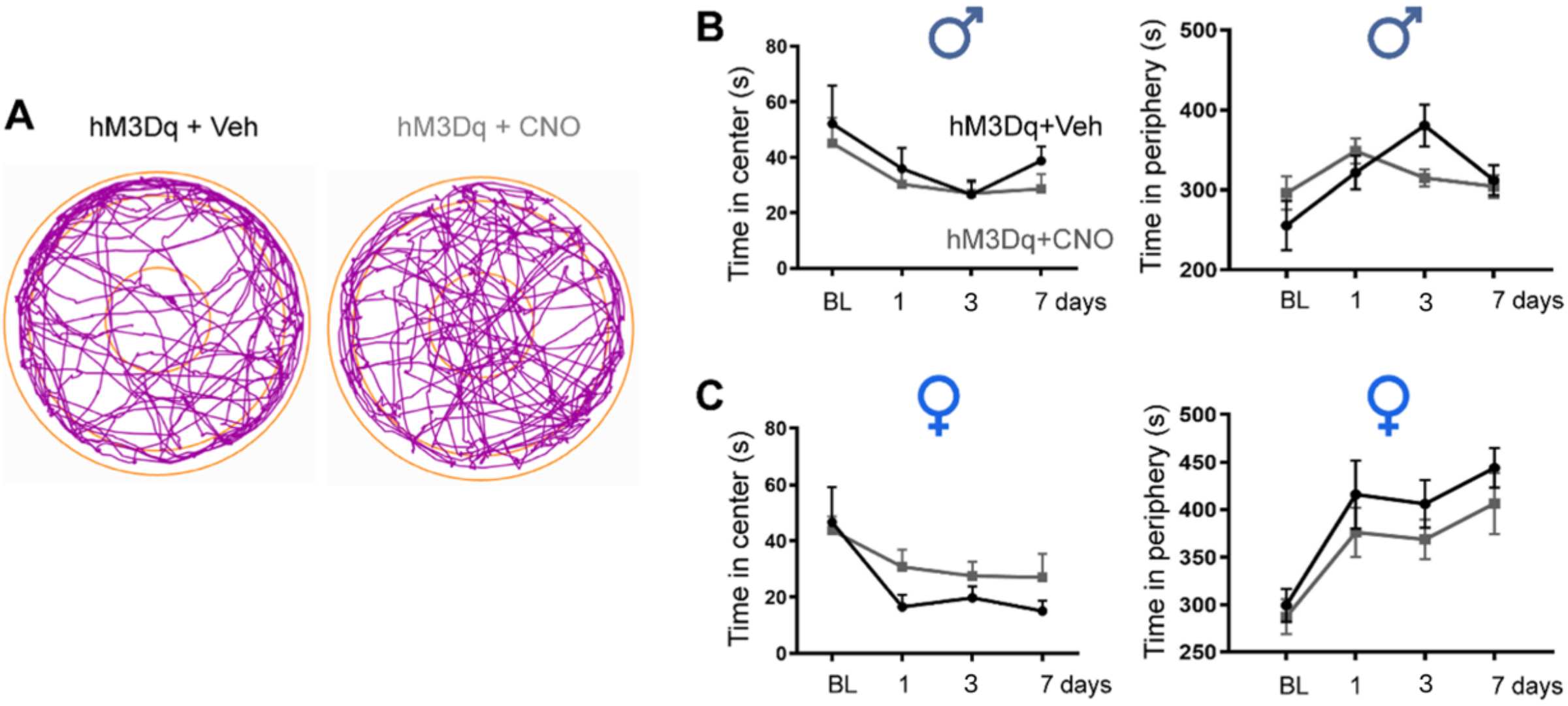

Fig. S3: Effects of microglia Gq activation on thigmotaxis after pMCAO.

(**A**) Track plots showing the movement of two mice in the open field arena during 10 minutes of exploration. (**B**) Time spent in the center vs. the periphery of the arena as a measure of anxiety, evaluated at baseline (BL) and at days 1, 3 and 7 after stroke (male mice). (**C**) Same analysis for female mice. Microglia Gq activation (light gray) had no effect by itself (BL) and did not significantly alter stroke-induced thigmotaxis in either sex.

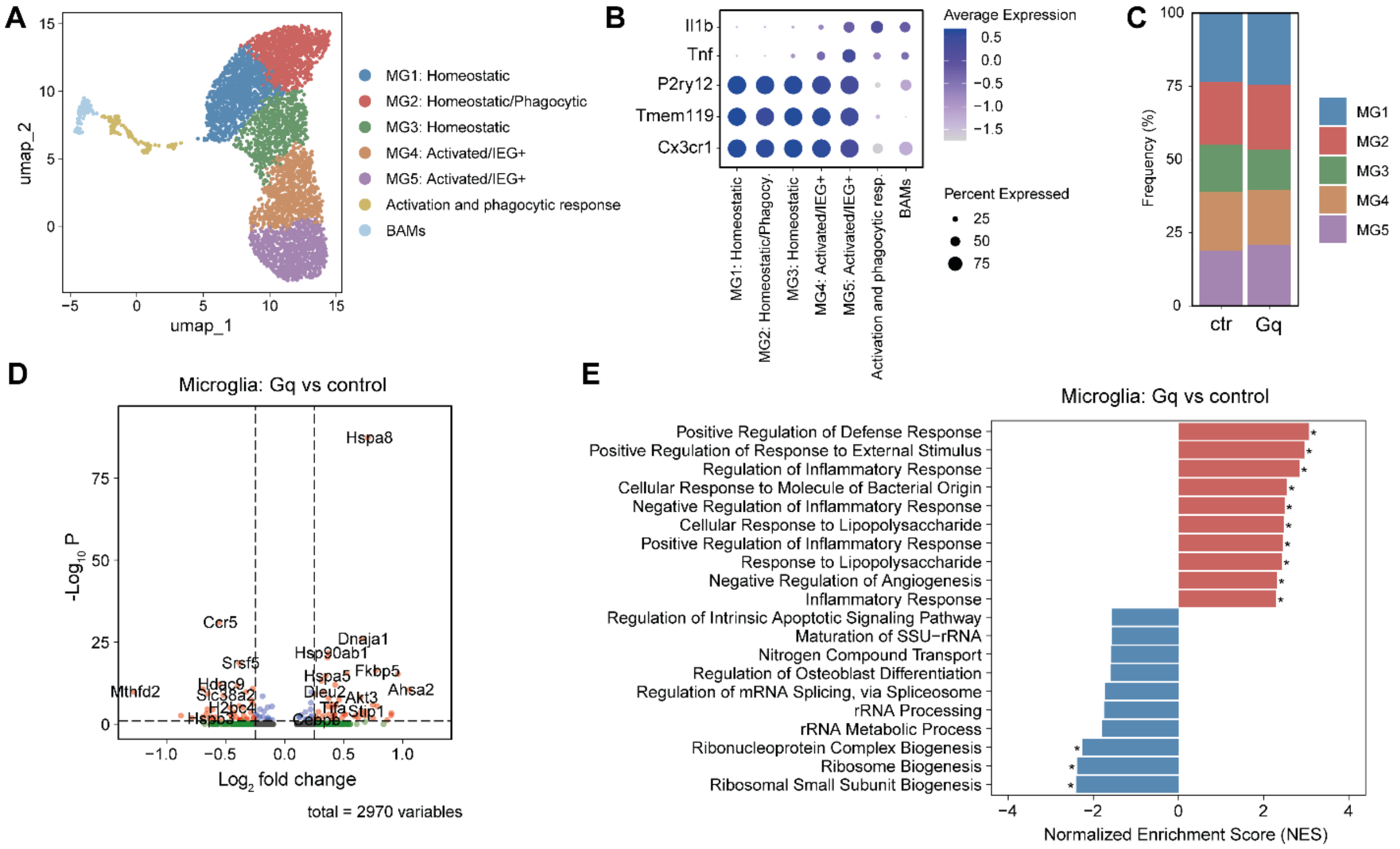

Fig. S4: Gq activates defense responses in microglia.

(**A**) UMAP-based clustering of microglia from Gq mice and controls (N = 2 , 2) after 3 daily CNO injections (no stroke). (**B**) Microglia clusters (MG1-5) express the marker genes P2ry12, Tmem119 and Cx3cr1. Clusters MG4 and MG5 express the activation markers interleukin 1 (Il1) and tumor necrosis factor (Tnf). (**C**) Microglia Gq activation in vivo did not induce state switching. (**D**) Gq activation lead to significant upregulation of Heat Shock Protein Family A Member 8 (Hspa8) and Heat Shock Protein 90 Alpha Family Class B Member 1 (Hsp90ab1) which play key roles in chaperone-mediated autophagy. (**E**) Gene set enrichment analysis (GSEA) of microglia (Gq-activated vs controls). Asterisks next to bars indicate a significant adjusted P-value. MG, microglia; BAMs, border-associated macrophages.

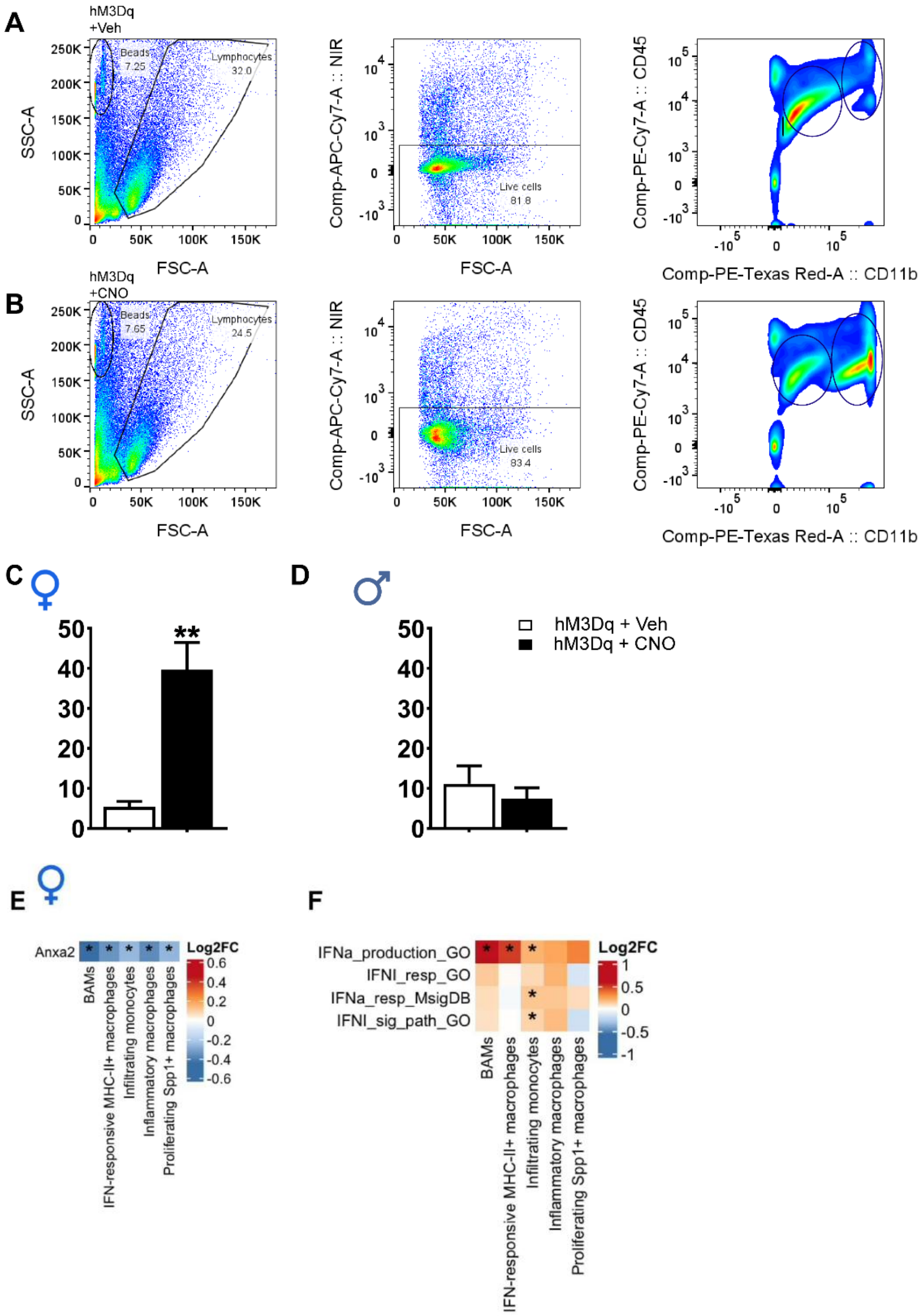

Fig. S5: Increased stroke-induced myeloid infiltration in female mice after Gq activation.

(**A-B**) Gating strategy for $CD45^{high}CD11b^{high}$ myeloid cells (Veh, n = 5 female mice; CNO, n = 7 female mice). (**C**) In female mice, microglia Gq activation leads to increased infiltration of myeloid cells into the ipsilateral hemisphere 7 days after stroke. (**D**) In male mice, microglia Gq activation has no effect on stroke-induced myeloid infiltration (Veh, n = 7; CNO, n = 8). (**E**) Gene set activity of myeloid subsets show reduced *Anxa2* in all female subsets, and (**F**) increased IFN production in border-associated macrophages (BAMs) and IFN-responsive MHCII macrophages subclasses. Data are shown as mean ± SEM. Significant differences indicated with * for $p < 0.05$, ** for $p < 0.01$.
